# A randomized controlled trial to evaluate the impact of pharmacist-led clinical interventions on the health-related quality of life among TB patients

**DOI:** 10.3389/fphar.2023.1171985

**Published:** 2023-05-24

**Authors:** Farman Ullah Khan, Faiz Ullah Khan, Muhammad Tahir Aqeel, Khezar Hayat, Jie Chang, Asim ur Rehman, Yu Fang

**Affiliations:** ^1^ Department of Pharmacy Administration and Clinical Pharmacy, Xi’an Jiaotong University, Xi’an, China; ^2^ Center for Drug Safety and Policy Research, Xi’an Jiaotong University, Xi’an, China; ^3^ Shaanxi Center for Health Reform and Development Research, Xi’an Jiaotong University, Xi’an, China; ^4^ Research Institute for Drug Safety and Monitoring, Institute of Pharmaceutical Science and Technology, Xi’an, China; ^5^ Faculty of Pharmacy, Hamdard University Islamabad Campus, Islamabad, Pakistan; ^6^ Department of Pharmacy, Faculty of Biological Sciences, Quaid-i-Azam University, Islamabad, Pakistan

**Keywords:** tuberculosis, patient-centered care, intervention, health-related quality of life, EQ-5D-3L

## Abstract

**Background:** The study was designed to evaluate the impact of pharmacist-led clinical interventions on the health-related quality of life among tuberculosis patients in Pakistan.

**Methods:** A randomized, controlled prospective study was carried out in a Pakistan Institute of Medical Sciences hospital tuberculosis (TB) control center. Participants who visited the TB center between September 2020 and December 2021 were randomly assigned to two clusters, the usual care group (UC group) *vs.* the intervention group (pharmaceutical care group), in a 1:1 ratio by a simple envelope technique. In the intervention group, a patient received centered care that encompassed informed decision-making, which can increase the quality of care and monitoring of adverse drug events. However, the control group received routine TB treatment at the hospital. The EuroQol-5D-3L instrument was used to assess the health-related quality of life (HRQoL) at the baseline and in the third and sixth months of the treatment time period.

**Results:** A total of 503 patients were eligible, of which only 426 patients were included in this study. At the end of the study, *n* = 205 of the patients in the intervention group and *n* = 185 of those in the control group were analyzed. In the intervention group, the EQ-5D-3L health utility score improved significantly (*p <* 0.001) (from the baseline mean ± SD, 0.40 ± 0.36, to 6 months of treatment, 0.89 ± 0.09, while in the control group from 0.42 ± 0.35 to 0.78 ± 0.27). In multivariate regression analysis, the variables that remained statistically associated (*p <* 0.001) with the HRQoL (unstandardized β [95% confidence interval]) of the control group were as follows: gender, female *vs.* male (-0.039 [-0.076 to -0.003]); body weight, less than 40 kg *vs.* more than 40 kg (-0.109 [-0.195 to -0.024]); patients with any comorbidity *vs.* without comorbidity (-0.136 [-0.252 to -0.020]); and smokers *vs.* non-smokers (-0.204 [-0.291 to -0.118]). The study did not find any statistically significant associations between the intervention group’s variables and the HRQoL.

**Conclusion:** Patient-centered care interventions led by pharmacists as part of care coordination enhanced the HRQoL for TB patients significantly. According to this study, clinical pharmacists should be included in the interdisciplinary clinical staff for TB patient management.

## 1 Background

Tuberculosis (TB), a severe, chronic lung disease, remains a major public health concern worldwide, particularly in low- and middle-income countries (Abiz et al., 2020; Khan et al., 2021). The World Health Organization (WHO) defines health as “full physical, psychological, and social welfare,” not just the absence of disease and disability (Saxena et al., 2001). The effect of TB on a patient’s health is considered essential because it can confront physical limitations and psychological, social, and economic constraints (Saleem et al., 2018). People who are suffering from TB have low self-esteem, a negative impression of their illness, and poor communication with their family and community members (Vecino et al., 2011; Iqbal et al., 2014). As a result, TB patients are challenged with physical problems, psychological stress, and economic issues (Zarova et al., 2018). More importantly, this lowers the health-related quality of life (HRQoL) of patients, which can also decrease the outcome of TB treatment (Jaber et al., 2016).

Pakistan ranks fifth among the 22 high TB-burdened countries (Aggarwal, 2019; Chen et al., 2021). In developing countries, particularly in Pakistan, very few studies have been carried out on the HRQoL of TB patients (Malik et al., 2018; Organization, 2021). Unfortunately, in most TB control programs, the quality of life of TB patients is neglected (Malik et al., 2018; Chen et al., 2021). Currently, TB control services are geared in the direction of enhancing the cure rate. Despite the fact that a positive cure rate is required, it does not alleviate TB patients’ physical, emotional, and social distress (WHO, 2020). In light of this, the WHO has realized that there needs to be better support for people with TB so that they can feel less distress, have a better quality of life, and get better results from their treatment. As a result, it advocates for “integrated patient-centered care and prevention.” This is very important for TB patients because it puts each patient’s rights, values, and needs at the center of TB control strategies (Jeremiah et al., 2022; Petersen et al., 2022). An ethical strategy for eliminating TB must be patient-centered and based on human rights. The social and personal conditions of the individual afflicted by TB must be given primary attention in addition to the urgent needs of medical therapy since TB patients encounter significant problems that go beyond the clinical aspects of the illness (Cocozza et al., 2020). According to recent findings, appropriately integrated patient-centered treatment can improve TB patients’ control (Kastien-Hilka et al., 2017a; Cocozza et al., 2020). Patient-centered care empowers patients to exercise their rights and fulfill their obligations while also improving their HRQoL through better knowledge of their health (Kastien-Hilka et al., 2017b; Yuen et al., 2021). However, little is known about whether the patient-centered care model would be helpful in improving the HRQoL of TB patients in Pakistan. The aim of this randomized, controlled trial was, thus, to assess whether pharmacist-led clinical interventions would improve the health-related quality of life of TB patients in Pakistan.

## 2 Methods

### 2.1 Study design

This randomized controlled prospective study was carried out in the tertiary-care hospital of the Pakistan Institute of Medical Sciences TB control center in Islamabad, Pakistan.

### 2.2 Study participant randomization

A single-blinded randomized controlled trial (RCT) was implemented, utilizing two parallel arms that were equally divided (in a 1:1 ratio using a simple envelope technique) between the intervention and control groups. The RCT has an intervention group, in which patients will be given pharmaceutical care + usual care, versus a usual care group, where participants will follow DOTS care according to the protocol of the TB control program and WHO (Organization, 2016). DOT by definition means observing TB patients to make sure they swallow each dose of anti-TB medication. This study is registered with Clinicaltrials.gov NCT04645836. The Standard Protocol Items: Recommendations for Interventional Trials guidelines were followed in this study. Participants who visited the TB center between September 2020 and December 2021 were included in this study. To search for the aspects that affect the HRQoL of TB patients, the data questionnaire was divided into two sections. The first section discusses trial variables based on a literature review, while the second section assesses the HRQoL using EQ-5D-3L and patient satisfaction with counseling using a pre-validated Urdu version questionnaire (Naqvi et al., 2019). A recruitment patient registration team referred interested participants for evaluating their eligibility and attaining informed consent. The process of randomization was conceded through a computer Research Randomizer, completed by the principal investigator. After that, the participants were randomly generated within sealed opaque envelopes. The envelopes were opened by the study participant allocation team. The pharmacist received all the pharmaceutical care group envelopes and counseled them in a private room at the TB control center, while DOTS supporters received usual care envelopes. The data collector’s team and the data analysis team that carried out outcome measurements, such as the quality of life and satisfaction with the intervention, remained fully blinded to the allocation of the control and intervention groups. The trial pharmacist was the primary contact person in case of possible problems during the trial. Still, even the contact person was entirely blinded to the preliminary outcome assessment to have fewer chances of bias.

#### 2.2.1 Inclusion and exclusion criteria

The study’s participants also included those who were 18 years of age or older and were getting self-administered TB treatment. At the baseline, all patients who agreed to a pharmacist’s counseling session were enrolled in the trial. The pharmacists engaged in the research study must be registered with the Pakistan Pharmacy Council and have a valid Category A license granted by the Pharmacy Regulatory Authority of Pakistan. Patients under the age of 18, those with extrapulmonary TB, and those having difficulties in writing or speaking Urdu were excluded from the study. Furthermore, patients with incorrect contact information, transfer out, and lack of follow-up were excluded from the study analysis.

### 2.3 Pharmaceutical care intervention

In addition to their regular treatment, all patients in the intervention group received individualized patient care from a clinical pharmacist. A healthcare paradigm known as “centered care” puts the patient at the center of all decisions. This method takes patients’ individual needs, wants, and values into account while including them as active partners in their own care. Direct patient monitoring, lifestyle modification education, and counseling are among the services provided.

#### 2.3.1 Patients’ pharmaceutical care and interventions

1) After the documentation of baseline data, patient care interventions were implemented. Pharmaceutical care was provided during follow-up visits to collect and prepare care plans for every TB patient. The intervention was divided both into non-pharmacological and pharmacological categories. Non-pharmacological treatments included information on appropriate drug use, while pharmacological interventions included medication addition or modification.
2) Both verbal and written methods of educating patients had been used. The booklet provides information on medication administration and lifestyle modification.
3) The pharmacist identified disease-related drug problems and made management recommendations during treatment.
4) Participants received a daily SMS text and weekly phone calls for anti-TB medicine intake and medication refill visit reminders.

##### 2.3.1.1 Phases of interview

People in the intervention group were checked on at the start of the study, after 3 months, and after 6 months. The patient’s QoL was inspected at each follow-up visit. The first interview was conducted at the baseline before initiating the intensive phase of anti-tuberculosis treatment. The second interview was performed within 2 weeks of switching to the continuation phase, and the third interview was conducted at the end of 6 months. A separate room was set aside for patient counseling and interviews for the intervention group. The pharmacist in the intervention group had no access to or involvement in the care of the control group patients. To ensure a moral impression, all participants who visited the TB clinic after screening obtained an instructional booklet with information on preventative treatment for family members.

### 2.4 Usual care group

Both intervention and control groups received the clinic’s usual services, including education session and drug prescription by a physician, as necessary, and the phone contact number of the clinic. The hospital staff provided usual care to these groups; physicians, a nurse, and pharmacy technicians were all involved in the management. They were treated according to the hospital’s clinical standards, including assessment, medication management by hospital pharmacy technician staff, and a normal patient follow-up at the TB healthcare center. The pharmacist did not intervene on their behalf; however, these patients were given advice based on their needs, and they were not obligated to attend any pharmacist counseling sessions.

### 2.5 European quality of life scale (EQ-5D five-dimensional questionnaire)

The health-related quality of life is a phrase that characterizes a person’s overall life satisfaction and wellbeing concerning their health and medical treatment. The HRQoL takes into account multiple aspects that determine physical, emotional, and social health, including pain, mobility, emotions, and social connections. EQ-5D-3L, designed by the EuroQol group, is frequently used as an HRQoL questionnaire that provides a single health status index value and a basic description (Dion et al., 2004). EQ-5D-3L consists of a descriptive system, which includes a five-dimensional/question set of health mobility, self-care, usual activity, pain, and anxiety, in which each dimension can further be classified into three levels of severity responses: level 1, no problem; level 2, some problem; and level 3, extreme problem. The EQ-5D-3L questionnaire also includes a 20-cm health meter and a visual analog scale (VAS), on which the respondents’ current self-health state is recorded on two distinct end points of a graduated (0–100) scale, with 100 being the best imaginable health state and 0 being the worst imaginable health state. EuroQol offered the Urdu (national language of Pakistan) version of EQ-5D-3L, and the study was also registered with an EuroQol ID: 34816. The scores for each dimension can be added together to provide a health status; a score of 1 on each dimension (11111) indicates perfect health. By using country-specific value sets, each patient’s five health states can be summed up into a single health utility value. The EuroQol-specific value sets are not yet documented for the Pakistani general population. Therefore, EQ-5D-3L was scored using values from a general population survey conducted in the United Kingdom in 1995, which had previously been used in an early survey in Pakistan (Saleem et al., 2018; Shahid et al., 2018). Feedback from patients regarding pharmacist counseling was identified through a pre-validated Urdu version patient satisfaction feedback questionnaire.

### 2.6 Ethical considerations

The Ethical Research Board of the Pakistan Institute of Medical Sciences (PIMS) (F.1–1/2015/ERB/SZABMU/359) was also approved by Xi’an Jiaotong University (XJTU), Health Science Center Biology Scientific and Research Ethics Committee (2019–1257). The respondents were also asked to give their written and verbal consent to take part in this study.

### 2.7 Statistical analysis and sample size

For continuous data, mean standard deviation was used, whereas for categorical variables, data were presented in percentages and frequencies. The normality of the data distribution was checked using the Kolmogorov–Smirnov test. To test for statistical significance among patient variables, one-way ANOVA tests were used for the control and intervention comparison within the groups (EQ-5D-3L index score and EQ-VAS score). A *p*-value of less than 0.05 was considered significant. All the variables that were included in preliminary analyses of multivariates were examined to verify that the tolerance value variance inflation factor and homogeneity of variance were not violated. In multivariate analysis, independent variables having *p* < 0.2 in univariate analysis were included in the analysis. Statistical Package for Social Sciences (SPSS) version 26 software was used to analyze the data obtained. We assumed a 10% difference in the primary outcome (Mishra et al., 2017), an alpha of 0.05, and a power of 80%, thus requiring a minimum sample size of 385 patients for both groups. However, considering the potential for losses to follow-up to be 10%, the intended sample size was 213 subjects per group over a 6-month study time period; the final sample size was 426 patients for both groups.

## 3 Results

### 3.1 Patient enrolment

**FIGURE 1:**
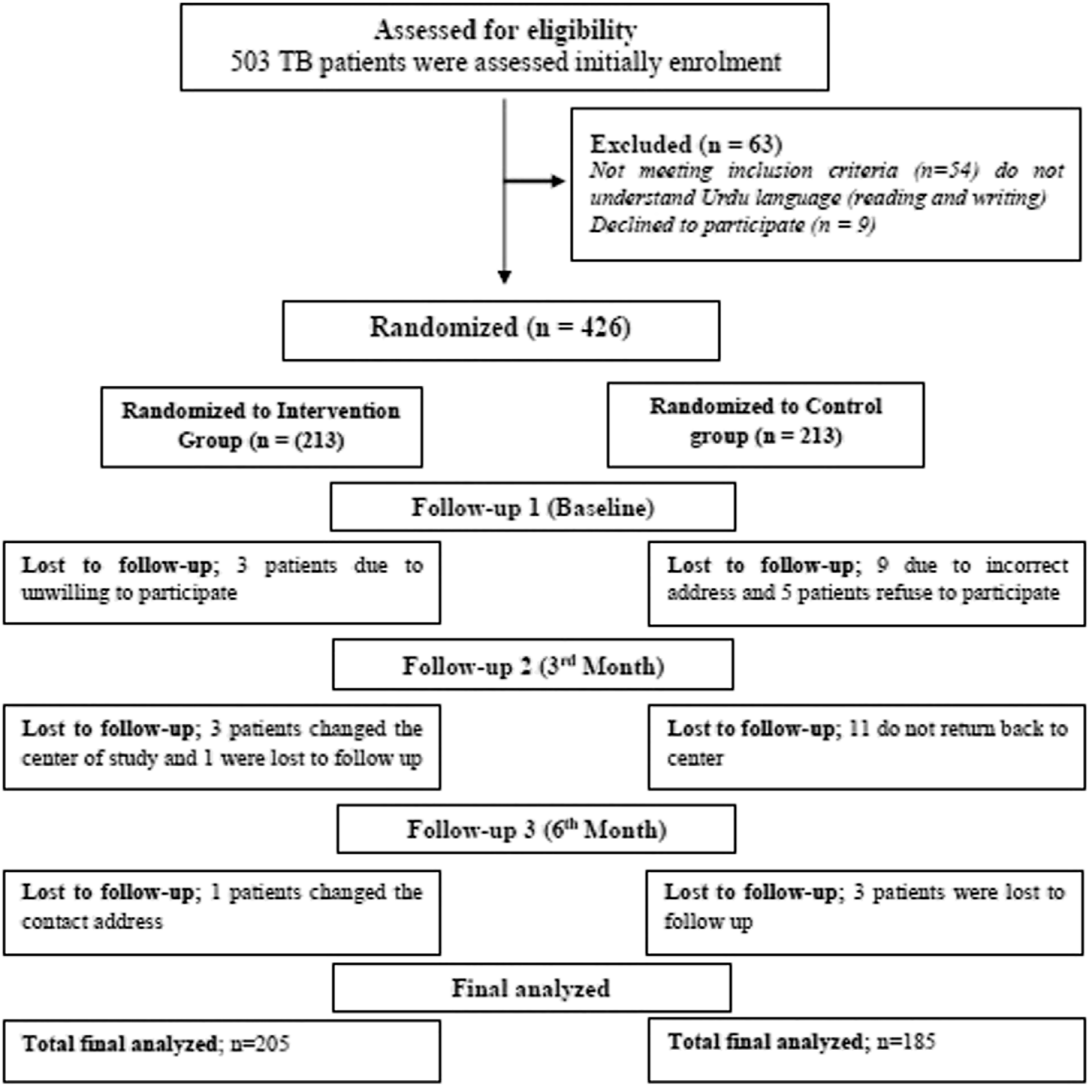
Flowchart of the participants enrolled in the RCT (intervention and control groups).

After they were found to be eligible, 426 of the 503 patients were randomly split into two groups: the intervention group with 213 patients and the control group with 213 patients. At the end of the study, *n* = 205 of the patients in the intervention group and *n* = 185 in the control group were included in the analysis. As indicated in Figure 1, the control group had 28 patients who dropped out, whereas the intervention group had eight patients who dropped out.

### 3.2 Patient demographic characteristics

The age group of 31–50 years represented almost 40.2% of the participants in the study, followed by 18–30 years (30.5%) and >51 years (29.2%), with male patients dominating the study (54.3%). More than half of the participants (233, 59.7%) had no formal education, while the remaining participants had intermediate level education (67, 17.1%) and primary level education (73, 18.7%). Out of the total, 71, 18.2%, patients in the study were employed. More than half (69.4%) of them had urban residences. The majority of study participants (70.5%) were from low-income families. Diabetes (5.8%), hypertension (7.1%), and hepatitis C, co-infected with TB, were the most common comorbidities in the overall sample of 16.1% of patients, while 21.2% of patients were under 40 kg; the percentages of non-smokers and current smokers were 76.2% and 23.8%, respectively (Table 1).

**TABLE 1:** Patients’ characteristics of the study groups, control *vs.* intervention.

Variable	Intervention group, *n* = 205 (%)	Control group, *n* = 185 (%)	*p*-value
**Age (years)**			0.51
18–30	65 (31.8)	57 (30.8)	
31–50	77 (37.6)	77 (43.2)	
>51	63 (30.8)	51 (27.6)	
**Gender**			0.12
Male	123 (60)	89 (48.1)	
Female	82 (40)	96 (51.9)	
**Qualification**			0.31
No education	121 (59)	112 (60.5)	
Primary level	40 (19.5)	33 (17.8)	
Intermediate level	34 (16.6)	33 (17.8)	
Master level	4 (2.0)	5 (2.7)	
Religious	6 (2.9)	2 (1.1)	
**Education category**			0.31
Illiterate	121 (59)	112 (39.5)	
Literate	84 (41)	73 (60.5)	
**Employment status**			0.06
Employed	31 (15.1)	40 (20.8)	
Unemployed	174 (84.9)	145 (78.4)	
**Locality**			0.06
Urban	150 (73.2)	121 (65.4)	
Rural	55 (26.8)	64 (34.6)	
**Income**			
Low	148 (72.2)	130 (70.3)	0.06
Intermediate	44 (21.5)	51 (27.6)	
High	13 (6.3)	4 (2.2)	
**Body weight**			0.07
<40 kg	37 (18)	46 (24.9)	
≥40 kg	168 (82)	139 (75.1)	
**Type of comorbidities**			0.28
Diabetes	14 (6.8)	9 (4.9)	
Hypertension	13 (6.3)	12 (6.5)	
Hepatitis	5 (2.4)	1 (0.5)	
Other			
**Comorbidities**			0.08
No	162 (83.9)	165 (89.2)	
Yes	33 (16.1)	20 (10.8)	
**Cigarette smoking**			0.22
Smoker	39 (19)	42 (22.7)	
Non-smoker	166 (81)	143 (77.3)	
**Reported to center**			0.09
Delayed	83 (40.5)	97 (47.6)	
Not delayed	122 (59.5)	88 (52.4)	

Kilogram (kg).

### 3.3 The comparison of HRQoL scores among the usual and intervention groups

The patient’s QoL from usual care was evaluated from the first to the second follow-up (mean ± SD standard deviation, 0.42 ± 0.35 to 0.57 ± 0.29) and from the second to the third follow-up (0.57 ± 0.29 to 0.78 ± 0.27) (Table 2).

**TABLE 2:** Comparison of HRQoL scores among the control and intervention groups.

Group	Pharmaceutical care group (EQ-5D utility score, mean ± SD)	Usual care group (EQ-5D utility score, mean ± SD)	*p*-value
			
Month 0 baseline	0.40 ± 0.36	0.42 ± 0.35	0.65
After 3 months	0.72 ± 0.25	0.57 ± 0.29	0.00
After 6 months	0.89 ± 0.09	0.78 ± 0.27	0.00
	EQ-VAS score	EQ-VAS score	
Month 0 baseline	45.3 ± 28.9	47.9 ± 28.1	0.36
After 3 months	71.7 ± 21.1	62.1 ± 25.4	0.00
After 6 months	85.5 ± 8.36	79.6 ± 16.6	0.00

There was no significant difference between both groups at the baseline. The result showed that after the intervention in the second and third follow-up visit, the intervention group had significantly higher mean scores than the control group of EQ-5D-3L utility. In the second follow-up visit in the pharmaceutical care group, patients’ EQ-5D utility score increased to mean ± SD 0.72 ± 0.25, while in control group participants, the EQ-5D utility score increased to 0.57 ± 0.29; there were statistically significant differences in the mean and standard deviation scores (*p <* 0.001). In the final follow-up visit, the pharmaceutical care group patients’ utility score mean (SD) increased to 0.89 ± 0.09, while in the usual care group, the score was 0.78 ± 0.27 (*p <* 0.001).

#### 3.3.1 Health-related quality of life domain comparisons between the control and intervention group

When compared to the control group, the intervention group’s mean QOL domain scores improved significantly (*p <* 0.001). The worst affected domains were the psychological domain in both groups. After pharmacist counseling, in the intervention group, more improvement was seen in the psychological domain (*p <* 0.00). In the first follow-up visit, significant differences were present in the mobility mean and standard deviation score of the intervention group (1.46 and 0.54) and the control group (1.57 and 0.56) (*p <* 0.04). In the second follow-up visit, there was a significant difference between EQ-5D-3L utility scores of the intervention and control groups (*p* < 0.05). In the third follow-up assessment in three domains, self-care (*p <* 0.00), usual activities (*p <* 0.00), and psychological domain (*p <* 0.00), there were significant improvements in the intervention group compared to the UC group (Table 3).

**TABLE 3:** Change of mean and standard deviation scores, and EQ-5D-3L and EQ-5D-VAS scores.

First follow-up	Domain	Intervention group (mean ± SD)	Control group (mean ± SD)	*p*-value <0.05
	Mobility	1.46 ± 0.54	1.57 ± 0.56	0.04
	Self*-*care	1.91 ± 0.72	1.86 ± 0.64	0.49
	Usual activities	1.86 ± 0.66	1.88 ± 0.64	0.84
	Pain	1.98 ± 0.64	1.93 ± 0.56	0.41
	Anxiety*/*depression	2.12 ± 0.76	2.03 ± 0.70	0.23
**Seco**nd **follow-up**				
	Mobility	1.08 ± 0.27	1.23 ± 0.42	0.00
	Self*-*care	1.26 ± 0.54	1.85 ± 0.68	0.00
	Usual activities	1.45 ± 0.60	1.83 ± 0.65	0.00
	Pain	1.55 ± 0.63	1.70 ± 0.59	0.04
	Anxiety*/*depression	1.32 ± 0.53	1.76 ± 0.74	0.00
**Third follow-up**				
	Mobility	1.02 ± 0.16	1.02 ± 0.16	0.89
	Self*-*care	1.02 ± 0.16	1.49 ± 0.65	0.00
	Usual activities	1.16 ± 0.39	1.42 ± 0.54	0.00
	Pain	1.17 ± 0.39	1.22 ± 0.41	0.26
	Anxiety*/*depression	1.20 ± 0.40	1.54 ± 0.73	0.00

**FIGURE 2:**
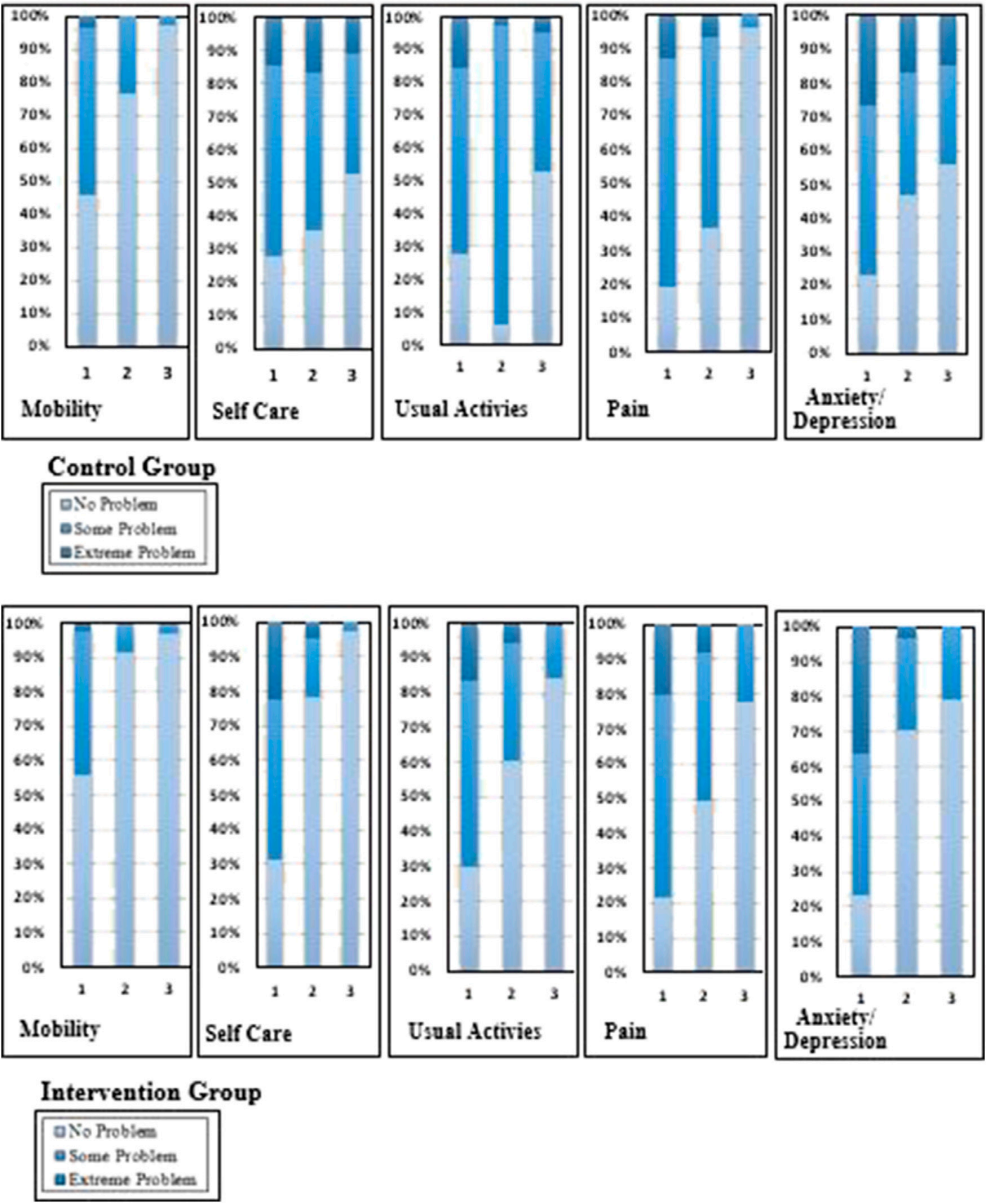
Change in the percentages of the quality of life in five domain scores of the intervention and control groups.

At the first follow-up visit, more than half the respondents reported moderate or severe difficulties with mobility, usual activities, pain, and anxiety/depression. In the control group, severe problems existed in self-care (14.6%), usual activities (15.7%), pain (13%), and anxiety (26.5%). By the second follow-up visit, 16.8% of patients reported extreme problems with anxiety and depression, 14.1% in usual care, 17.8% in self-care, and 6.5% in the pain category. At the third follow-up visit, no patients in the control group reported extreme mobility problems, while 14.6% reported problems with anxiety, 8.6% in self-care, and 2.7% reported extreme problems in usual activities (Figure 2).

In the intervention group, extreme problems were experienced by TB patients in self-care (22.4%), usual activities (16.6%), pain (20%), and anxiety (36.1%). By the second follow-up visit, 8.8% reported extreme problems with pain, 5.9% in usual care, 4.9% in self-care, and 3.4% in the anxiety and depression category. At the third follow-up visit in the intervention group, no patients reported extreme problems; 1% of severe problems were identified in usual activities (Figure 2).

### 3.4 Regression analysis of the HRQoL by patients’ characteristics

A univariate logistic regression was used to quantitatively analyze the interactions of the study variables that had close associations with the TB-related HRQoL. A good fit model for the multivariate linear regression was determined (*F* = 8.66, *p <* 0.001, and adjusted R^2^ = 0.255). In the univariate logistic regression, the variables in control group participants that were associated with the HRQoL included the following: age groups, 31–50 *vs.* 18–30 (unstandardized β [95% CI]; *p*-value) (0.27 [-0.013 to 0.067]; *p* = 0.18) and >51 *vs.* 18–30 years -0.03 [-0.060 to 0.001]; *p* = 0.04); gender, female *vs.* male (-0.04 [-0.080 to -0.001]; *p* = 0.04); there was a significant difference in the study outcome between patients with a body weight less than 40 kg compared to those with body weight greater than 40 kg (-0.185 [-0.273 to -0.097]; *p *= 0.00), with a comorbid condition *vs.* without a comorbid condition (-0.212 [-0.336 to -0.087]; *p* = 0.00), cigarette smokers *vs.* cigarette non-smokers (-0.226 [-0.315 to -0.137]; *p* = 0.00), and with the those reported to the center within 30 days *vs.* those reported after more than 30 days (0.124 [ 0.080 to 0.168]; *p* = 0.00) (Table 4).

**TABLE 4:** Difference in the HRQoL through patients’ characteristics of the intervention group using the univariate linear regression.

Variable	Control group EQ-5D-3L index (univariate linear regression)		Intervention group EQ-5D-3L index (univariate linear regression)	
	Unstandardized β [95% CI]	*p*-value	Unstandardized β [95% CI]	*p*-value
Age (years)				
31–50 *vs.* 18–30	0.27 [-0.013 to 0.067]	0.18	0.006 [-0.007 to 0.020]	0.36
>51 *vs.* 18–30	- 0.03 [-0.060 to 0.001]	0.04	-0.017 [-0.016 to 0.003]	0.15
Gender				
Female *vs.* male	-0.04 [-0.080 to -0.001]	0.04	0.007 [-0.007 to 0.020]	0.32
Qualification				
Literate *vs.* illiterate	-0.019 [-0.059 to 0.022]	0.36	-0.002 [-0.011 to 0.016]	0.72
Employment status				
Unemployed *vs.* employed	0.018 [-0.067 to 0.030]	0.45	-0.006 [-0.025 to -0.012]	0.50
Locality				
Urban *vs.* rural	-0.035 [-0.119 to 0.048]	0.40	-0.617 [-1.197 to -0.037]	0.03
Income				
Low *vs.* high	0.011 [-0.076 to 0.098]	0.80	0.021 [ -0.008 to 0.050]	0.15
Intermediate *vs.* high	-0.012 [-0.057 to 0.032]	0.58	0.001 [ -0.016 to 0.017]	0.95
Body weight				
<40 kg *vs.* ≥40 kg	-0.185 [-0.273 to -0.097]	0.00	0.021 [-0.013 to 0.056]	0.18
Comorbidities				
Yes *vs.* no	-0.212 [-0.336 to -0.087]	0.00	-0.028 [-0.064 to 0.007]	0.12
Cigarette smoker				
Smoker *vs.* non-smoker	-0.226 [-0.315 to -0.137]	0.00	-0.007 [-0.040 to 0.0.027]	0.69
Reported center				
Not delayed *vs.* delayed	0.124 [ 0.080 to 0.168]	0.00	0.002 [-0.025 to 0.029]	0.87

Univariate analysis for significant variations with *p* < 0.2 in the HRQoL was included in multivariate regression analyses.

In multivariate regression analysis, the factors that remained statistically associated (unstandardized β [95% confidence interval]; *p*-value) with the control group were as follows: gender, female *vs.* male (-0.039 [-0.076 to -0.003]; *p* = 0.03); body weight less than 40 kg *vs.* more than 40 kg (-0.109 [-0.195 to -0.024]; *p* = 0.01); patients with any comorbidity *vs.* without comorbidity (-0.136 [-0.252 to -0.020]; *p =* 0*.*02); and smokers *vs.* non-smokers (-0.204 [-0.291 to -0.118]; *p* = 0.00) (Table 5).

**TABLE 5:** Difference in the HRQoL through patients’ characteristics of the control group using multiple linear regression coefficients.

Variable	Control group EQ-5D-3L index (multivariable linear regression model)		Intervention group EQ-5D-3L index (multivariable linear regression model)	
	Unstandardized β [95% CI]	*p*-value	Unstandardized β [95% CI]	*p*-value
Age (years)				
18–30 *vs.* >51	0.009 [-0.033 to -0.051]	0.66	Not applied	-----
31–50 *vs.* >51	-0.015 [-0.046 to 0.016]	0.32	-0.007 [-0.016 to 0.002]	0.14
Gender				
Male *vs.* female	-0.039 [-0.076 to -0.003]	0.03	Not applied	-----
Qualification				
Illiterate *vs.* literate	Not applied	-----	Not applied	-----
Employment status				
Employed *vs.* unemployed	Not applied	-----	Not applied	
Locality				
Rural *vs.* urban	Not applied	-----	-0.031 [-0.060 to -0.001]	0.08
Income				
Low *vs.* high	Not applied	-----	0.018 [-0.011 to 0.048]	0.21
Intermediate *vs.* high			Not applied	
Body weight				
<40 kg *vs.* ≥40 kg	-0.109 [-0.195 to -0.024]	0.01	0.015 [-0.019 to 0.049]	0.39
Comorbidities				
Yes *vs.* no	-0.136 [-0.252 to -0.020]	0.02	-0.024 [-0.060 to 0.011]	0.17
Cigarette smoker				
Smoker *vs.* non-smoker	-0.204 [-0.291 to -0.118]	0.00	Not applied	-----
Reported center				
Delayed *vs.* not Delayed	-0.032 [-0.068 to -0.005]	0.09	Not applied	-----

CI (confidence interval), univariate analysis with *p* < 0.2 is considered for the fit model multivariate linear regression for the control group analysis with *F* = 8.66, *p <* 0.001, and adjusted R^2^ = 0.255, and for the intervention group model with *F* = 2.30, *p* < 0.04, and adjusted R^2^ = 0.055. Collinearity (variance inflation factor = 10) and tolerance value < 0.1.

In the univariate logistic regression (Table 4), the variables of the intervention group participants that were associated with the HRQoL included the following: age group >51 *vs.* 18–30 (-0.017 [-0.016 to 0.003]; *p* = 0.15), residency urban *vs.* rural (-0.617 [-1.197 to -0.037]; *p* = 0.03), income status of the intervention group participants (0.021 [-0.008 to 0.050]; *p* = 0.15), body weight less than 40 kg *vs.* more than 40 kg (0.021 [-0.013 to 0.056]; *p* = 0.18), and patients with any comorbidity *vs.* without comorbidity (-0.028 [-0.064 to 0.007]; *p = 0.12*). A good fit model for the multivariate linear regression was determined (*F* = 2.30, *p < 0.04*, and adjusted R^2^ = 0.055), while in multivariate regression analysis, the factors were not statistically associated with the HRQoL (Table 5).

### 3.5 Patient satisfaction

The majority of respondents (92.2%) stated that they were able to obtain advice from a pharmacist without difficulty. A total of 78.1% of patients agreed that they had gained TB-related knowledge based on their requirements. The majority of patients in the intervention group (83.7%) expressed satisfaction with regards to their concerns about medication. A majority of them (97.5%) seemed willing to recommend pharmacists’ counseling to others and suggested that this program be offered in pharmacies throughout the localities in the country. Moreover, 63.9% of patients have said they are prepared to pay for counseling in the future (Table 6).

**TABLE 6:** Feedback from patients regarding pharmacist counseling.

Patient satisfaction feedback regarding pharmacist counseling	Response	*n* (%)
Were you able to get counseling without any difficulty?	Yes	189 (92.2)
	No	16 (7.8)
Were you able to obtain the knowledge you required?	Yes completely	160 (78.1)
	Yes, to some extent	25 (12.1)
	No, I did not get	20 (9.8)
Did you find the pharmacist helpful in resolving your questions?	Very helpful	171 (83.4)
	Somewhat helpful	24 (11.7)
	Not helpful	10 (4.8)
What is your opinion about the time duration of pharmacist counseling?	More time should be given	60 (29.2)
	Appropriate time was given	139 (67.8)
	My time was wasted	6 (2.9)
Will you recommend getting counseling from pharmacists to others?	Yes	200 (97.5)
	No	5 (2.5)
In your opinion, should this service be offered by pharmacies in your locality?	Yes	205 (100)
	No	0 (0)
Are you willing to pay for this counseling service?	Yes	131 (63.9)
	No	74 (36.1)
How would you rate your satisfaction with pharmacist counseling?	Very satisfied	147 (71.7)
	Satisfied	40 (19.5)
	Uncertain	13 (6.3)
	Not satisfied	5 (2.4)

## 4 Discussion

Since health is a basic human right and a crucial global societal objective that is essential for human needs, it is important to improve the HRQoL, even in disease conditions (Pinto et al., 2017). TB management is very complicated that involves several drugs and a lengthy duration of treatment. These aspects have a substantial impact on the patients’ quality of life (Qiu et al., 2020). The EQ-5D-3L questionnaire was used in this RCT to assess the HRQoL and its important determinants in TB patients. To the best of our knowledge, this is the first RCT for evaluating the improvements in the HRQoL in TB patients from a low-income country, Pakistan. The involvement of a clinical pharmacist service in standard treatment resulted in an enhanced HRQoL in 6-month follow-up visits. EQ-5D-3L (mean 0.40–0.89) and EQ-VAS (mean 45.3–85.4) improved with the intervention provided by the pharmacist. Findings similar to our study were reported in previous studies (Mishra et al., 2017; Saleem et al., 2018; Ali et al., 2019; Munsour et al., 2020). According to the research study, the quality of life of the intervention group increased much more, following educational counseling (Awaisu et al., 2012; Howyida et al., 2012; Kh et al., 2014). One of the main reasons for this improvement in the HRQoL was the patient-centered care that may have helped patients get rid of their symptoms and improve their ability to accept anti-TB treatment. The present study’s findings supported a similar argument that empowering patients through centered care positively affects the HRQoL (Khachadourian et al., 2020). These findings recognize the significant role that a pharmacist performs as a member of a multidisciplinary team in improving the quality of life and medical services of TB patients.

Consistent with prior research studies (Bauer et al., 2015; Kisaka et al., 2016), our findings suggest that EQ-5D and EQ-VAS scores significantly increase as the treatment progresses. According to earlier research (Dujaili et al., 2015; Singh et al., 2017), the greatest improvement in EQ-5D scores was seen in the first 2 months and 6 months after treatment. In this study, the baseline EQ-5D utility score of TB patients was similar to the previous study conducted by Saleem et al. (2018) and was lower than 0.70 according to the study by Awaisu et al. (2012), showing differences in absolute values between studies (Awaisu et al., 2012; Saleem et al., 2018). The results of EQ-5D index scores vary depending on the value set (“tariff”) because each population may show different preferences for various health conditions (Van Hout et al., 2012). Unfortunately, research studies on the EQ-5D-3L utility score for TB patients are still rare; consequently, the findings of this study on utility scores across different health statuses might serve as a reference for such analyses. In this study, the highest increase in EQ-5D scores was recorded within the first 2 months of treatment; this result is similar to that previously reported by Dujaili et al. (2015) and Saleem et al. (2018). Physical, mental, and social problems are common among TB patients, due to prolonged therapy and the infectious nature of the disease (Zarova et al., 2018; Aggarwal, 2019). This finding is consistent with previous research studies (Singh et al., 2017; Aggarwal, 2019). The psychological domain has reported more problems than the physical, self-care, and pain categories; in addition, the results are consistent with those of a past study (Sartika et al., 2019). At the initiation of treatment, anxiety and depression were found in 36.1% of intervention group patients and 26.5% of the control group patients. The point of concern is that 14.6% still indicated anxiety and depression at the end of treatment in the control group. This is comparable to previous studies conducted in Pakistan, which reported that 46% and 55.5% of TB patients have anxiety and/or depression after treatment, respectively (Husain et al., 2008; Saleem et al., 2018). Another study reported that the reasons for the low HRQoL even after treatment were the long treatment period, isolation from their family, and low socioeconomic status (Sulehri et al., 2010). This finding highlights the critical importance of assessing TB patients’ mental health and providing appropriate counseling to those who require it the most.

Similar to other research studies, in this study, we also sought to understand the factors that explained the difference in the quality of life scales (Eq.(5D)–(3L)) (Louw et al., 2016; Banerjee et al., 2019). Educating people may result in a better ability to cope with TB infections and an increase in the awareness about illness control and prevention, which will increase the HRQoL (Wu et al., 2009; Chizimba, 2012). Memory problems, anxiety and depression, and economic difficulties all are identified as contributing factors to a lower HRQoL (Chen et al., 2021; Febi et al., 2021). Based on the analysis, male patients with TB had an improved HRQoL compared to female patients, and these findings are comparable with previous research studies (Samuel et al., 2022). Female patients are the most neglected population in society, possibly due to the high levels of stigma and discrimination against them in Pakistan (Habib et al., 2021). As a result, gender is an important social predictor of health, and inequalities in gender-related HRQoL parameters must be considered (Izhar et al., 2021). In this study, in the control group, smoking was identified to interact with the trend of the HRQoL of TB patients. Just as the previous studies proposed, smoking suppresses the immune system, resulting in higher bacillary loads, which can aggravate disease symptoms and decrease physical health improvement with treatment (Banerjee et al., 2019; Magis-Escurra et al., 2021). The study results propose that comorbidity illnesses significantly affect the HRQoL. The relative reduction in the QoL among those with comorbidities is similar to the previous studies (Johansson et al., 2013; Butterly et al., 2023).

The present study’s findings supported similar arguments that empowering patients through centered care positively affects the HRQoL (Khachadourian et al., 2020). These findings recognize the significant role that a pharmacist performs as a member of a multidisciplinary team in improving the quality of life and medical services of TB patients. The HRQoL of TB patients improved significantly in the intervention group. This shows the involvement of the clinical pharmacist in TB care, leading to positive effects in patient care and the HRQoL across all five domains in all patients in the intervention group. The findings of this study are similar to those of the previous studies (Munsour et al., 2020; Abubakar and Atif, 2021). This shows that a pharmacist’s involvement in TB care increases patients’ HRQoL across all five domains in all patients in the intervention group. Most patients found counseling easy and acknowledged the pharmacist’s support; they were satisfied with the answers they received and sought out more counseling opportunities. Our findings are in line with those of other studies (AlQarni et al., 2019; Naqvi et al., 2019). A significant proportion of participants were willing to pay for the service, and the level of satisfaction is consistent with the findings from studies conducted in Pakistan and Saudi Arabia (AlShayban et al., 2020; Khan et al., 2022). Pharmacists should provide patients with precise, clear, and relevant information about their medications. The findings of this study point to the importance of clinical pharmacist services in the TB health sector in Pakistan.

### 4.1 Limitations

This study’s notable advantage was that it was the first randomized controlled trial to examine how EQ-5D-3L scores of individuals changed in Pakistan, a country where TB is prevalent. Moreover, treatment and consultation were provided free of charge to our study patients. This interventional study outcome will help in establishing the significance of fulfilling a pharmacist-led educational intervention in TB care settings to improve patients’ self-care practices and treatment outcomes. There are, however, several limitations to this study that should be noted. First, there is the probability of a selection bias among TB participants since in this study only those participants who were willing to take part and the study condition of fluency in the national language Urdu limited the range of possible participants. A significant number of patients who visit the medical facility are from diverse towns and are only able to communicate effectively in their local language. Therefore, they have been unable to participate in the study. All participants were ensured that their refusal to be involved in the study would have no impact on their routine treatment so that they would not feel forced to play a part in the trial. Second, we applied an EQ-5D-3L health utility value based on the UK general population, although it has been conducted previously in Pakistan (Saleem et al., 2018; Habib et al., 2021). To provide a more accurate evaluation of the HRQoL in future research studies, a health utility value based on the general population of Pakistan should be established.

### 4.2 Policy implications

It is necessary to include the patient perspective when prescribing anti-TB medications. When patients are involved in treatment, they become vital participants in the control and eradication of TB (Organization, 2014). A patient-centered strategy will increase their social support and advocacy about the disease and treatment, which will help in improving patient satisfaction, HRQoL, and treatment outcomes, benefiting both the patients and society. Even though the present exercise is limited in low- and middle-income countries, this approach needs to be adopted. Therefore, the policymakers should initiate its implementation and documentation on a priority basis.

## 5 Conclusion

This study appears to be the first RCT in Pakistan addressing the HRQoL of TB and enhancing a pharmacist’s role in patient-centered care in collaboration with the TB control team. Patient-centered care interventions led by pharmacists, as a part of care coordination, enhanced the HRQoL for TB patients significantly. Most prominently, these findings show that the addition of pharmacists as group members in providing patient-centered care in low-income countries can positively support the TB-related HRQoL. Low-income countries should follow the patient-centered care approach. The findings of this study point toward the importance of clinical pharmacist services in the TB health sector in developing countries.

## Data Availability

The raw data supporting the conclusion of this article will be made available by the authors, without undue reservation.
